# The asymmetric impact of global warming on US drought types and distributions in a large ensemble of 97 hydro-climatic simulations

**DOI:** 10.1038/s41598-017-06302-z

**Published:** 2017-07-19

**Authors:** Shengzhi Huang, Guoyong Leng, Qiang Huang, Yangyang Xie, Saiyan Liu, Erhao Meng, Pei Li

**Affiliations:** 1 State Key Laboratory Base of Eco-Hydraulic Engineering in Arid Area, Xi’an University of Technology, Xi’an, 710048 China; 2 Joint Global Change Research Institute, Pacific Northwest National Laboratory, College Park MD, USA

## Abstract

Projection of future drought is often involved large uncertainties from climate models, emission scenarios as well as drought definitions. In this study, we investigate changes in future droughts in the conterminous United States based on 97 1/8 degree hydro-climate model projections. Instead of focusing on a specific drought type, we investigate changes in meteorological, agricultural, and hydrological drought as well as the concurrences. Agricultural and hydrological droughts are projected to become more frequent with increase in global mean temperature, while less meteorological drought is expected. Changes in drought intensity scale linearly with global temperature rises under RCP8.5 scenario, indicating the potential feasibility to derive future drought severity given certain global warming amount under this scenario. Changing pattern of concurrent droughts generally follows that of agricultural and hydrological droughts. Under the 1.5 °C warming target as advocated in recent Paris agreement, several hot spot regions experiencing highest droughts are identified. Extreme droughts show similar patterns but with much larger magnitude than the climatology. This study highlights the distinct response of droughts of various types to global warming and the asymmetric impact of global warming on drought distribution resulting in a much stronger influence on extreme drought than on mean drought.

## Introduction

Drought is considered as one of the most costly natural disaster due to the devastating impacts on agriculture, infrastructure, industry, and tourism^1^. In recent years, major droughts have occurred across different regions of the world^2^, including Australia^3^, China^4^, the Amazon^5^, Sahel^6^ and North America^7, 8^ with huge impacts on water resource management and ecosystem productivity^9–11^. In order to mitigate the losses and manage water shortages accompanying droughts, it is necessary to investigate future evolution of droughts in a consistent and comprehensive manner. Previous studies show increases in the frequency and severity of droughts at the global scale under various climate change scenarios^2, 12–16^. However, discrepancy often exists in the projected change in specific characteristic of drought at the regional/local scale, which could be attributed to the difference in the drought type, drought index, spatial-temporal scales, hydro-climate model/data and our incomplete knowledge on the underlying mechanisms behind drought. For example, Sheffield *et al*.^17^ found little change in drought over the past 60 years when the changes in available energy, humidity and wind speed are considered in calculating the Palmer Drought Severity Index (PDSI)^18^, in contrast to other conclusions^2, 19^. Regionally, in the US Great Plains, the PDSI suggests more intense drought in the future across the Great Plains^2, 20, 21^, while weak drying is projected based on soil moisture projections^22^.

It is commonly accepted that drought can be grouped into meteorological, agricultural, hydrological and socio-economic drought categories^23–26^. Meteorological drought is defined as precipitation deficit while soil moisture and low river flow are often termed as agricultural drought and hydrological drought, respectively. Because different drought indices measuring various drought types contrast sharply^2, 17^, it is important to compare these drought types to understand why their results are different. Previous studies have investigated future changes in drought based on the anomaly of precipitation^27, 28^, soil moisture^29, 30^ or streamflow/runoff^16, 31, 32^, separately. To date, investigation of meteorological, hydrological and agricultural drought in a consistent manner is rare^26, 33^, not to mention its concurrence which could exert more severe effects than any other single drought type in terms of impacts^34, 35^. Recently, Zhang *et al*.^36^ has conducted a pioneering work by investigating the change pattern of four types of drought in India and examined drought impacts on wheat yield with a focus on the historical period. What’s more, a single drought event can be characterized by its duration and intensity, while several metrics can be used to measure the probabilistic distribution of droughts in a specific period, e.g., the total occurrences, mean and extreme duration/intensity and etc. Changes in drought with different duration and intensity have distinct implications for drought management and adaptations. Sheffield *et al*.^13^ investigated three classes of drought duration (i.e., short-term, medium term, and long-term) but only considering the soil moisture based drought from limited raw climate model simulations. To date, most of previous investigations mainly focused on the mean of droughts, leaving the changes in drought of various types under different categories and the extremes under-investigated.

Recently, the Paris agreement explicitly asks for an assessment of the impacts of 1.5 °C global warming above preindustrial levels^37, 38^. However, very few studies have reported scenarios consistent with the 1.5 °C target, especially on the droughts. Here, we link regional droughts to global temperature rise and, specifically, assess the response of droughts to the 1.5 °C target to provide scientific basis on mitigation and adaptation measures. We focus our investigation in the conterminous United States (CONUS) where droughts have resulted in average annual damage up to $6–8 billions^1^. In this study, we try to investigate the drought of various types rather than examining the discrepancy arising from the formulation of a specific drought index. Specifically, we use a large ensemble of 97 1/8 degree hydro-climate model simulations to investigate future changes in the meteorological, agricultural, and hydrological droughts. By this, we attempt to explore the possible uncertainty in the projected droughts from the perspective of drought type, climate model and emission scenario, which is critical to inform policy-makings for adaptions. Specifically, we examine the following scientific questions: 1) How will drought change in terms of frequency, duration and intensity in response to different levels of global warming? 2) Are there robust patterns in the changes in meteorological, agricultural, hydrological drought and its concurrence? 3) Will the impacts of global warming on drought of various types and their probabilistic distributions be asymmetric? In other words, will drought change pattern be similar between the mean and extreme of the investigated drought types?

## Results

### Historical drought and model validation

**Figure 1:**
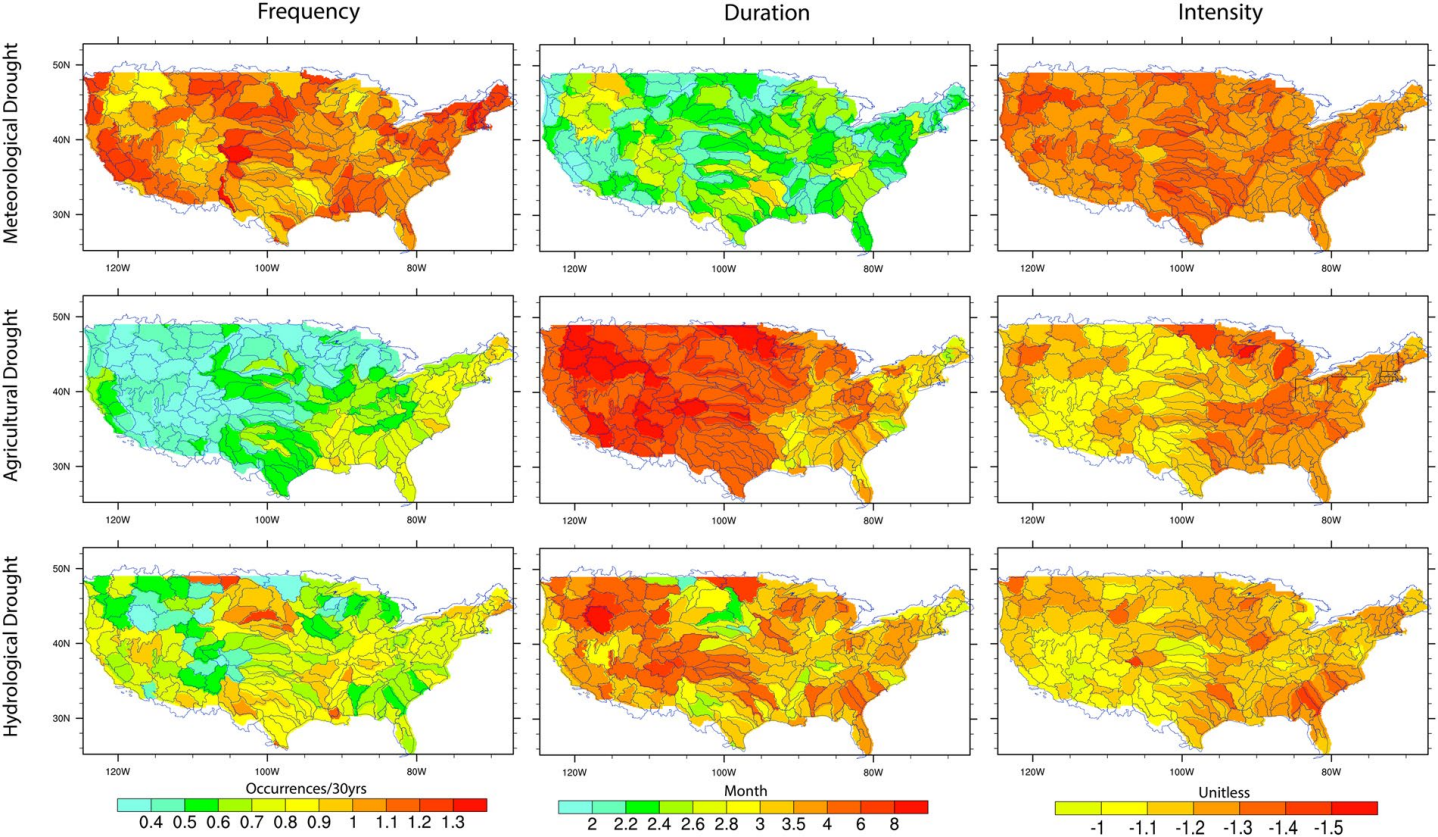
Spatial distribution of frequency (occurrences/30yrs), duration (months) and intensity (unitless) of meteorological, agricultural and hydrological droughts during 1971–2000. Meteorological drought is based on observed precipitation while hydrological and agricultural droughts are based on VIC simulated runoff and soil moisture driven by the observed climate, respectively. The frequency is referred to the number of drought occurrences for the given period while duration and intensity are for the climatology mean of those drought events. Figure was created by NCAR Command Language^81^.

Figure 1 shows the spatial distribution of frequency, duration and intensity of the three drought types based on observed precipitation and Variable Infiltration Capacity (VIC) model simulations (i.e., OBS-VIC, see methods for detail). That is, meteorological drought is based on observed precipitation while hydrological and agricultural drought are based on the simulated runoff and soil moisture by the VIC model driven with observed climate^39^, respectively. Distinct spatial patterns are found in the frequency, duration and intensity among the three drought types due to the role of land surface processes in regulating the response of agricultural and hydrological droughts to climate variability and change^40^. Specifically, soil moisture conditions, although correlated with precipitation at monthly timescales, could exhibit nonlinear responses to precipitation at shorter timescales^23^. In general, more frequent meteorological and hydrological droughts are found but with smaller durations than agricultural droughts. Meteorological droughts exhibit the largest intensity followed by agricultural and hydrological droughts across much of the country.

**Figure 2:**
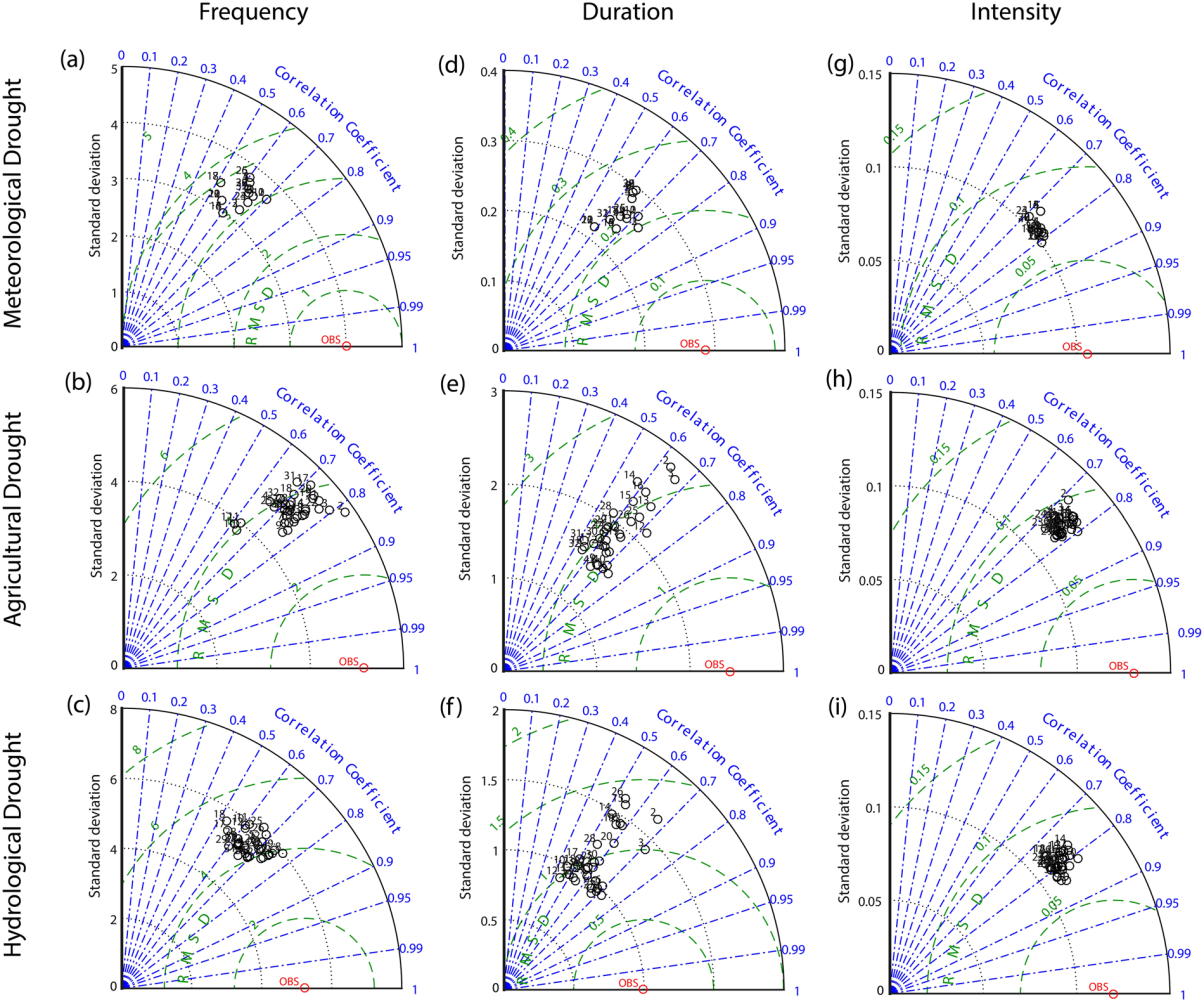
Taylor-diagram on the performance of CMIP5-VIC simulations in reproducing the frequency, duration and intensity of meteorological, agricultural and hydrological droughts by OBS-VIC during 1971–2000. The CMIP5-VIC refers to the bias-corrected CMIP5 climate and VIC simulations driven by bias-corrected CMIP5 climate. Black circles denote the simulations driven by CMIP5-VIC combinations with the numbers indicating the simulations driven by the climate model identified by the ID numbers in Table S1. Figure was created using software MATLAB 2015a (http://www.mathworks.com/).

Future changes in droughts are based on bias-corrected CMIP5 climate and VIC simulations driven by bias-corrected CMIP5 climate (i.e., CMIP5-VIC, see methods for details). Before our investigation of future changes in droughts, we first examine the validity of CMIP5-VIC simulations in reproducing historical meteorological, agricultural and hydrological droughts by OBS-VIC over CONUS. Instead of examining the general drying trend as in previous climate model validations^41^, we specifically investigate the validity in reproducing the frequency, duration and intensity of the three drought types. Figure 2 shows the Taylor diagram between CMIP5-VIC and OBS-VIC (i.e., the patterns in Fig. 1) in terms of frequency, duration and intensity of the three drought types. The spatial correlations between CMIP5-VIC and OBS-VIC range from 0.5 to 0.8 for most models, indicating the reasonable performance of the bias-corrected CMIP5-VIC simulations in capturing historical drought events. Model performance varies among the three drought types (i.e., meteorological, agricultural and hydrological droughts) and depends on the drought feature (i.e., frequency, duration and intensity) and metric (e.g., the spatial correlation, standard deviation and model range). As for the frequency, meteorological drought has lower model range compared to agricultural and hydrological droughts, indicating the better performance in simulating meteorological drought. However, the spatial correlation of simulated meteorological drought with observation is lower compared to agricultural and hydrological droughts, indicating the worse model performance in simulating meteorological drought. As for the duration and intensity, the model performance in simulating meteorological drought is higher than agricultural and hydrological droughts, as indicated by the lower model range and similar magnitude of spatial correlation. Larger model ranges are found in simulating drought duration followed by frequency and intensity, while wider ranges in simulating hydrological and agricultural droughts than meteorological drought are observed. The ability of bias-corrected CMIP-VIC simulations in reproducing historical drought patterns leads to higher confidence in projecting future droughts. Such validations have great implications for climate change impact assessment on droughts in which the adopted bias-correction technique is mainly used for reducing the bias in the climatology mean and/or quantile distributions^42^.

### Distinct response of regional drought of various types to global warming

**Figure 3:**
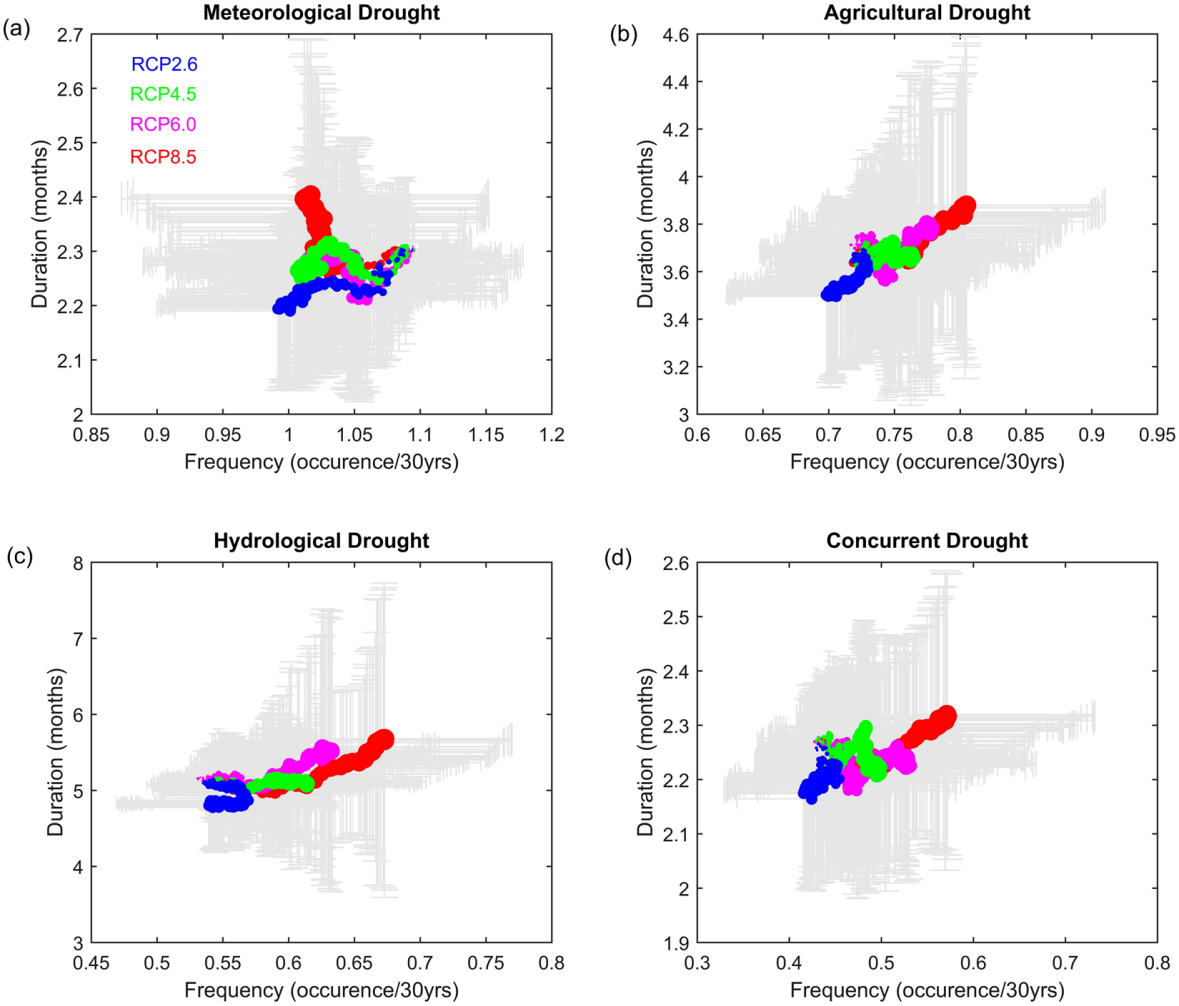
Changes in relations between US mean frequency and duration of (**a**) meteorological, (**b**) agricultural, (**c**) hydrological and (**d**) concurrent droughts by CMIP5-VIC simulations under RCP8.5, RCP6.0, RCP4.5 and RCP2.6 scenarios. Dots represent the multi-model ensemble mean values while error bar is for the one standard deviation to show the model ranges. The size of dots are scaled by global warming amount to characterize the regional changes in drought frequency and duration relations with global warming. For example, larger dots indicate larger global warming amounts or later future period given that global mean temperature exhibits an evident increasing trend with time, although there is small variation at the interannual scale. Figure was created using software MATLAB 2015a (http://www.mathworks.com/).

Figure 3 shows the evolution of US mean frequency and duration of meteorological, agricultural, hydrological and concurrent droughts based on CMIP5-VIC simulations under four Representative Concentration Pathway (RCP) scenarios (i.e., RCP8.5, RCP6.0, RCP4.5 and RCP2.6). Here, we show the changes with global mean temperature rather than the time, mainly because we attempt to link regional droughts with global temperature rise which are informative for mitigation and adaptation strategies. Changes in drought with time can be found in the Supplementary Figure S1. The US mean frequency of meteorological drought is decreasing with global warming, independent of emission scenarios. In contrast, agricultural and hydrological droughts are projected to become more frequent. This is reasonable given the fact that meteorological drought is based on precipitation deficit while agricultural and hydrological droughts are defined on the basis of soil moisture and runoff, respectively. Increase of precipitation does not necessarily result in increase of soil moisture and runoff, as the latter two are governed by several land surface processes such as evapotranspiration, soil storage and snow accumulation/melt^2, 43–45^. For example, evaporative demand would increase with global warming at a rate which is much faster than the increase of precipitation^2^. The deficit of precipitation minus evaporation (i.e., the available water) would result in less water in soils and rivers. Therefore, more agricultural and hydrological droughts are projected even if we have less meteorological drought. In general, increases in drought statistics are greatest under the highest emission scenario RCP8.5, and least under the lowest emission scenario RCP2.6. The differing responses of droughts to the same level of global warming demonstrate the importance to investigate droughts of various types in a consistent manner, instead of focusing one drought type as in previous studies. This also emphasizes the need to explicitly examine the changes in specific drought features which are neglected in global and regional scale studies. Associated with droughts are pronounced durations while decreasing durations of meteorological droughts are projected under RCP2.6 scenario. The most pronounced droughts are projected under RCP8.5 scenario in which global warming signal is much stronger than others.

**Figure 4:**
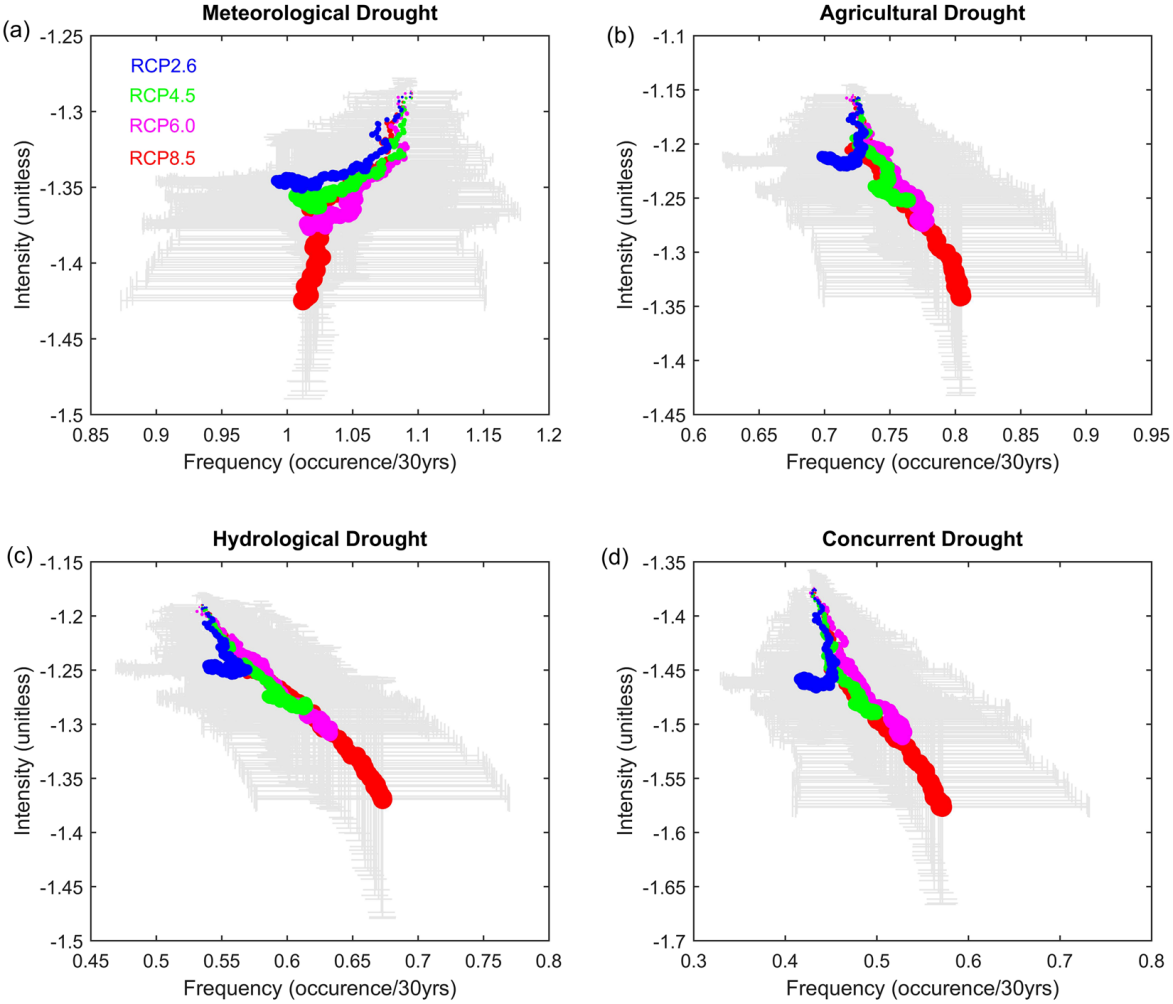
Same as Fig. 3 but for relations between drought frequency and intensity. Figure was created using software MATLAB 2015a (http://www.mathworks.com/).

In contrary to differing drought responses in terms of frequency and duration to global warming scenarios, more consistent changes in drought intensity are found. As shown in Fig. 4, droughts of all types are projected to become more severe with global warming independent of emission scenarios, although the inter-model ranges denoted by the error bars are considerable. The increase in drought severity could be attributed to increase in the vapor pressure deficits driven by higher temperatures and subsequent increase of atmospheric evaporative demand^20^. This also corroborates with previous findings demonstrating the role of temperature in strengthening drought severity in California^46^, Southern European^47^ and etc. Importantly, changes in drought intensity are found to scale linearly with global warming amount especially under RCP8.5 scenario at the 95% confidence level, implying that future drought intensities over CONUS can be derived directly from given level of global mean temperature rise. This has great implications for mitigation and adaptation strategies as drought projections often involve extensive computation cost in driving complex impact models with an ensemble of climate models. However, such relations are not evident under other emission scenarios. Changes in the concurrence of meteorological, agricultural, and hydrological droughts which could exert more severe effects than any other single drought type in terms of impacts generally follow the pattern of agricultural and hydrological droughts.

**Figure 5:**
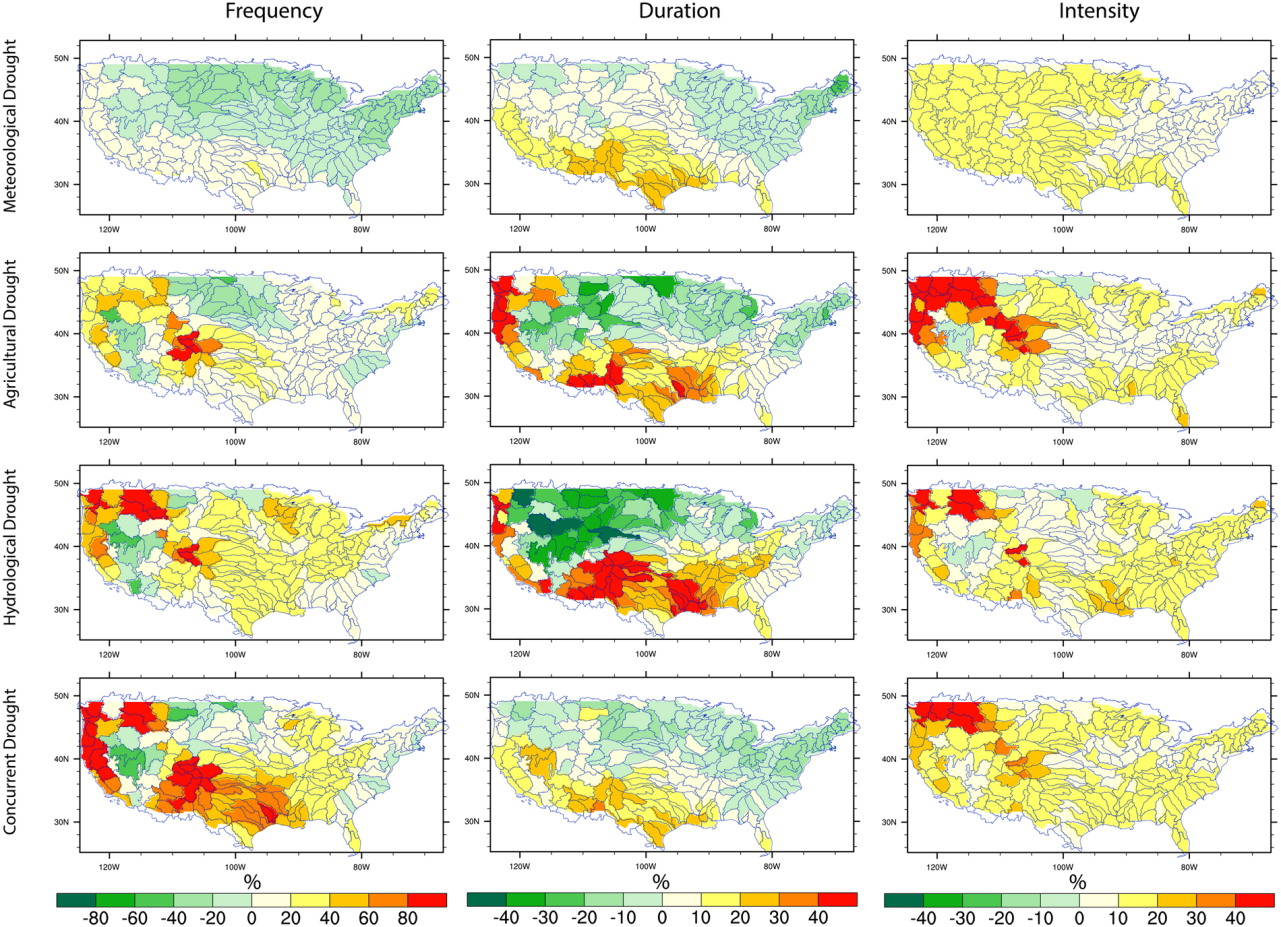
Multi-model ensemble mean changes (%) in the frequency, duration and intensity of meteorological, agricultural, hydrological and concurrent droughts in 1.5 °C global warming. The global mean temperature is first calculated for each 30-year time window. We select the first 30-year period when the +1.5C global warming is crossed for each climate model under each RCP. We then calculate the drought changes in the selected period corresponding to a 1.5C warming world for each model and the multi-model ensemble mean under each RCP is shown. Figure was created by NCAR Command Language^81^.

Spatially, most regions will experience decrease in meteorological drought frequency in the 1.5 °C warming world (Fig. 5). In regions that experience substantial snowfall in the winter and subsequent melt during the spring, higher temperature could further contribute to drying in spring/summer^48^, leading to increase in hydrological drought frequency. For example, agricultural and hydrological drought events show large percentage increase in Pacific Northwest and California where meteorological droughts are projected to decrease. Notably, the spatial patterns of changes in agricultural, hydrological and meteorological droughts are found to be weakly correlated. This indicates that agricultural and hydrological droughts cannot be directly determined from precipitation alone as models add information to the signal derived from precipitation^32^. Concurrent droughts experience substantial increases, in particular, in the Texas, California, Pacific Northwest and Colorado with relatively small changes over Northern Great Plains and eastern US. Much larger areas will experience decreasing duration of droughts, which is consistent among drought types except for larger magnitude of changes in agricultural and hydrological drought durations. Drought intensities are projected to increase in most of the country independent of drought types. In extreme case, agricultural and meteorological drought intensities are projected to increase by more than 40% in Pacific Northwest. Droughts are projected to increase the most in several hot spot regions which have experienced recent droughts (e.g., Texas, Pacific Northwest and California), highlighting the regions that are consistently projected to be more strongly affected by droughts in the future than other regions.

### Asymmetric impacts of global warming on drought distribution

**Figure 6:**
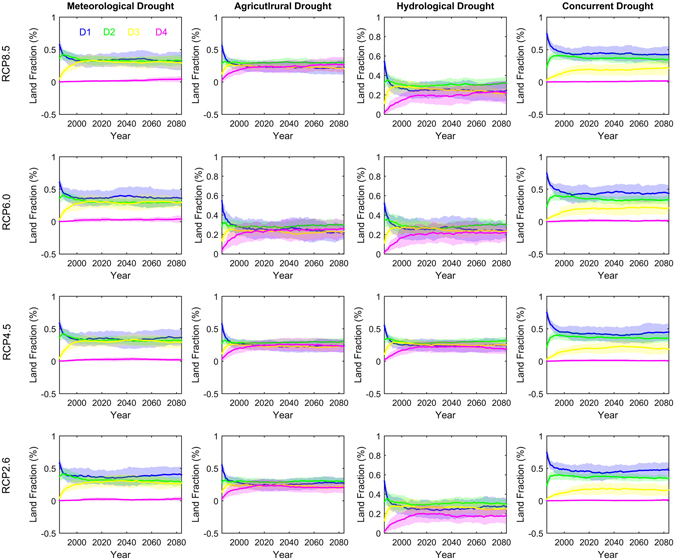
Fraction of US land area dominated by four categories of durations (i.e. D1, D2, D3 and D4) of meteorological (first column), agricultural (second column), hydrological (third column) and concurrent droughts (fourth column) under RCP8.5, RCP6.0, RCP4.5 and RCP2.6 scenarios. D1, D2, D3 and D4 are droughts with durations of 1~3, 4~6, 7~12, >12 months, respectively. The changes are calculated in a 30-year time window along 1971–2099 relative to 1971–2000. The year axis represents the 30-year period centered at that year. For example, the year 1985 indicates the period 1971–2000. Figure was created using software MATLAB 2015a (http://www.mathworks.com/).

A single drought event can be characterized by its duration and intensity, while certain number of droughts in a period can be measured by the climatological mean and distribution. Previous climate change impact studies mainly focused on the mean of droughts, leaving the changes in drought distribution under-investigated. Will impact of global warming on drought distribution be asymmetric? That is, will changes be similar between the mean and extreme droughts? Figure 6 shows the land fraction dominated by droughts in different duration bins. Here, we first calculate drought changes for each duration bin. We then compare the changes among the four duration bins and select the one which shows the largest change than other three bins as the dominant one (see methods). Generally, the drought structure exhibits an unstable pattern during the earlier study period (e.g., 1980s). The large variation during the earlier periods could be attributed to the fact that climate change impact signal is small in earlier periods as the changes are calculated relative to the reference period 1971–2000. With global warming, the signal tends to emerge with time and the defined drought structure become stable towards the end of 21^st^ century. Specifically, changes in meteorological and concurrent droughts with duration ranging from 1–3 months dominate ~30% of US’s lands, while the land fraction dominated by droughts longer than 12 months is less than 5%.Droughts longer than 12 months dominate up to 30% and 20% of US lands for agricultural and hydrological droughts, respectively, although durations of 4–6 months still exert the largest impacts. Importantly, land fractions dominated by different duration bins of droughts remain generally stable after 2020s for all drought types. Such pattern is consistent across the four emission scenarios although the magnitude would differ to some degree.

**Figure 7:**
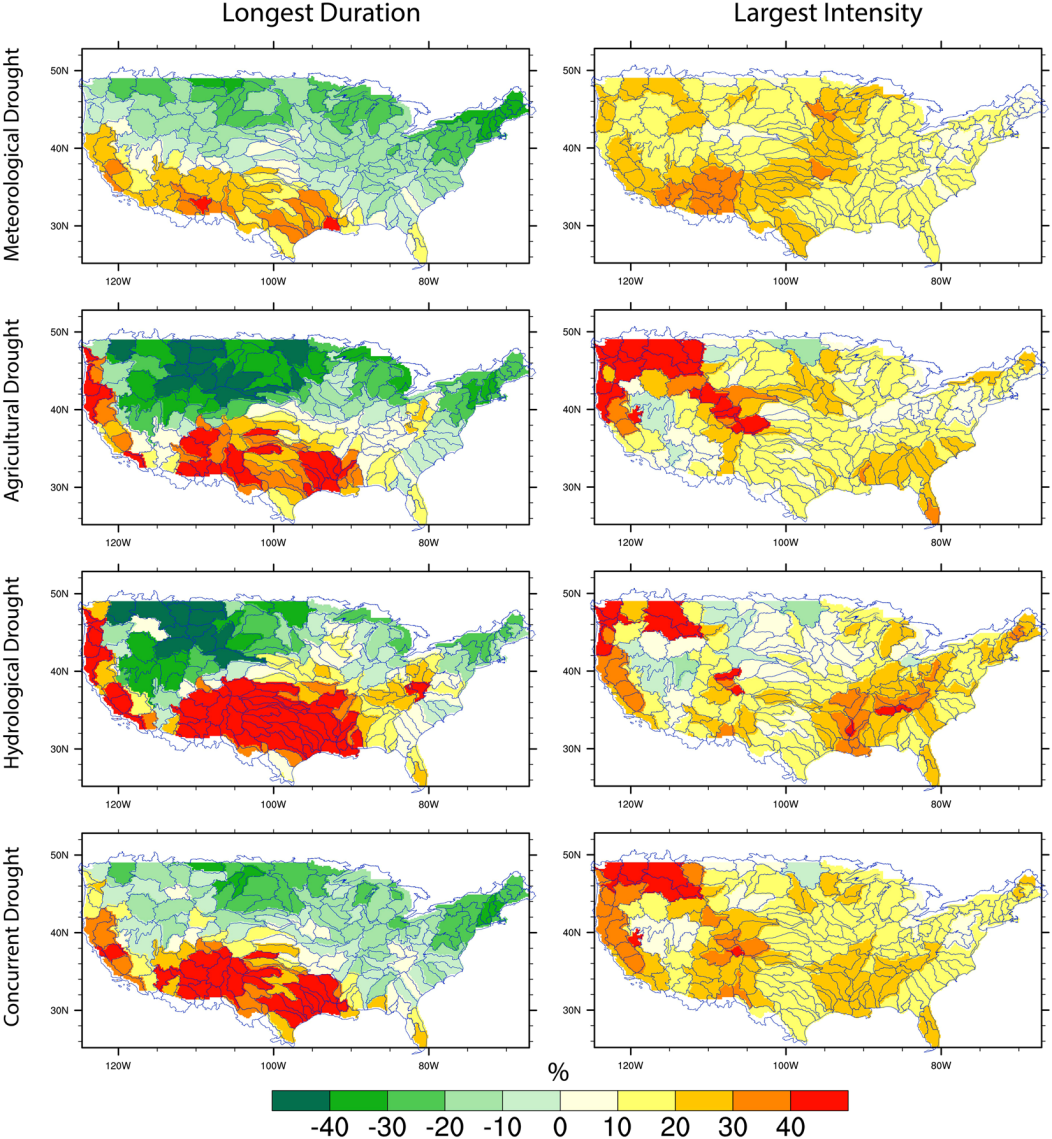
Multi-model ensemble mean changes (%) in the longest duration and largest intensity (i.e., the most negative drought intensity) of meteorological, agricultural, hydrological and concurrent droughts in 1.5 °C global warming. The global mean temperature is first calculated for each 30-year time window. We select the first 30-year period when the +1.5C global warming is crossed for each climate model under each RCP. We then calculate the drought changes in the selected period corresponding to a 1.5C warming world for each model and the multi-model ensemble mean under each RCP is shown. Figure was created by NCAR Command Language^81^.

We also examine the changes in extreme droughts in terms of longest duration and largest intensity. Indeed, changes in the longest drought can exert more profound impacts than the mean droughts. When averaged over the country, changes in the longest drought remain generally stable towards the end of 21^st^ century, consistent with the climatology mean but with larger uncertainty ranges (Supplementary Figures S2 and S3). However, it is evident that agricultural drought with the longest duration is projected to increase substantially from 15 months to 17 months at the end of 21^st^ century. The longer drought durations could be attributed to the greater increases in extreme precipitation than in the mean in a warming climate, which could lead to a reduction in the number of wet days and an increase in dry spell length (i.e., longer drought durations)^49–51^. Regionally, the spatial pattern of changes in extreme droughts follows that of mean droughts, but with much larger magnitude of changes (Figure 7). Cayan *et al*.^43^ showed that future droughts in southwest of US would be aggregated by reduced spring snowpack and late spring/summer soil moisture induced by warmer temperature. Our results are consistent with Cayan *et al*.^43^ identifying the southwest US as one of the hot spot regions with aggravated droughts in the future. The asymmetric change in drought distribution indicates that projected increases in droughts are mainly driven by changes in extreme droughts. Our results highlight the importance of examining drought distributions and its extremes given the uneven changing ratios as revealed in this study and the distinct implications of their impacts on water resource management and ecosystems.

## Uncertainty and Limitations

In this paper, we aim to complement previous studies by analyzing the most up-to-date ensemble of 97 hydro-climatic simulations under four emission scenarios. In particular, we analyze the changes in meteorological, agricultural, hydrological drought and its concurrence. We also investigate the changing patterns of drought frequency, duration and intensity. Inclusion of other drought properties such as the total span (occurrence × duration) is not within the scope of this study. By this, we aim to provide a comprehensive estimation of the related uncertainties, depending on drought indices, climate models and emission scenarios. Besides model biases as indicated by the validations in the result section, there are additional limitations to our general conclusions as certain influencing factors are not considered in the model, e.g., the effects of land use and land cover change^52^, human water use^53, 54^, vegetation dynamics^55^, groundwater and surface water interactions^56^. Especially, *Leng et al*.^57^ showed that irrigation water use can further exacerbate the low-flow conditions with climate warming. These local factors could interact and amplify/counteract the drought response to global warming. Hence, our results should mainly represent the first-order climate change impacts on droughts in CONUS.

In addition, we acknowledge that there are limitations in the analyzing approach. First, the period 1971–2000 was chosen as the present-day period. Hence, our results could be dependent on the reference period chosen for analyses. Second, it is commonly accepted that drought is a multi-scalar phenomenon^58, 59^. The time scale over which water deficits accumulate is also important^11^. Therefore, droughts identified by the three indices would depend on the fitting function and time scales selected in this study. The longer time scale SPI, SSI and SRI would be very similar due to the high correlation among precipitation, soil moisture and runoff. Whereas climate-based indices such as SPI describe the precipitation anomalies, we emphasize the importance to use hydrologic indices to describe the effects of climate anomalies on hydrologic conditions which are governed by land surface physical processes. However, only one impact model (i.e., VIC) is used in deriving agricultural and hydrological conditions. *Van Huijgevoort et al*.^32^ found that the spread of simulated hydrological drought among ten hydrological models is largest in regions with low runoff and smallest in regions with high runoff. Fourth, we focus on the changes of drought at the decade scale, ignoring the seasonality of drought which would change disproportionately, especially in snow dominated regions. Indeed, warmer temperatures tend to reduce snow cover through melting of the existing pack and increasing of the rainfall fraction to snowfall. Fifth, drought index has to rely on an assumption that samples follow a given probability density function. However, there is no single probability density function which is suitable for the whole world. In US, there are about 12% of sub-basins where the selected drought index can’t be fitted by the selected lognormal probability density function (Supplementary Figure S4). Therefore, caution should be exercised when interpreting the drought changes in these regions. Indeed, to fit one type of probability density across US characterized with distinct hydro-climatic regimes is difficult. This is one of the major challenges facing drought assessments as pointed out in our previous efforts on non-parametric drought index development^59, 60^. To focus on drought index is not within the scope of this study. Rather, we aim to provide an up-to-date comprehensive investigation of droughts of various types for the US domain. In addition, a drought event is defined when the drought index value is below -1 (corresponding to 6.7% of probability). Therefore, we have to acknowledge that the results may vary if a different drought threshold is used. Overall, although the revealed frequency-duration-intensity relations are dependent on the case study, impact model, drought index and drought threshold, and the model spread as quantified by the standard deviation is fairly large, it helps understand how regional drought of various types and its probabilistic distribution respond to various level of global warming amount.

## Conclusions

Potential changes in drought would have adverse impacts on water management, ecosystem and social-economy. However, measuring drought itself is non-trivial as various drought indices have been used for different types of drought. A single drought event can be measured by its duration and intensity, while certain number of droughts in a period can be characterized by the probabilistic distribution (e.g., mean and extreme drought). Uncertainties arising from climate models, emission scenarios and impact models also add up to the uncertainties^61, 62^, often making it hard to inform policy-makings for adaptions. This study provides a comprehensive investigation on regional droughts over CONUS in response to global warming. Specifically, we investigate the changes in meteorological, agricultural and hydrological droughts and the concurrence based on 97 high-resolution hydro-climatic model projections. We also conduct specific assessment of droughts in the 1.5 °C warming world as advocated in the recent Paris agreement to provide scientific basis for mitigation and adaptation strategies.

Our results show that historical drought patterns can be well reproduced by the model ensemble, although bias of various magnitudes exist in reproducing specific features of drought. With global warming, meteorological drought frequency will decrease in the future, while agricultural and hydrological droughts are projected to increase. Durations of meteorological, hydrological and agricultural droughts remain relatively stable when averaged over the country. On the contrary, the intensity of all drought types are projected to increase linearly under RCP8.5 scenario, indicating that future drought intensities over CONUS can be derived directly from a given level of global mean temperature rise in this scenario. The spatial structure of short, medium and long-term droughts will remain stable after 2020s while extreme droughts in terms of longest duration and intensity are projected to increase much faster than the climatological mean. Regionally, droughts are highlighted as one of the great challenges for several hot spot regions that are consistently projected to be more strongly affected by drought under the 1.5 °C warming world. Several of the regions are important agricultural areas (e.g., California and Midwest Corn Belt), on which global food production would critically depend in the future.

The projected increase in droughts with global warming represents a threat to humans and ecosystems in CONUS. The distinct response of droughts of various types to global warming indicates the importance of considering more than one type of drought in climate change impact assessments. This study emphasizes the asymmetric impact of global warming on the probabilistic distribution of droughts resulting in a much stronger influence on extreme droughts than mean droughts. This finding has important implications for informing water management strategies and adaption measures coping with high-impact drought extremes.

## Data and Methodology

### Hydro-climate projections

In this study, 97 climate model projections from the Coupled Model Intercomparison Project Phase 5 (CMIP5)^63^ under four Representative Concentration Pathway (RCP) scenarios (RCP2.6, RCP4.5, RCP6.0 and RCP8.5)^64^ are used (Supplementary Table S1) to capture the possible uncertainty range arising from climate models and emissions pathways. Compared to the CMIP3, the CMIP5 models have notable changes in model physics, resolution and greenhouse gas emission and land cover change scenarios. The CMIP5 climate model projections are statistically downscaled to 1/8 degree resolution and bias-corrected against the observed climate^39^ over CONUS using the bias-correction and spatial-downscaling approach (BCSD)^65^. Specifically, daily precipitation is first aggregated into monthly scale for both CMIP5 simulations and observations, based on which monthly scaling factors are calculated between the two during the overlap period^39^. Then, a historical daily precipitation pattern is resampled for a specific month and adjusted by the monthly scaling factor to match simulated monthly precipitation totals with observations, while preserving the observed wet-day fractions. By this, the bias-corrected daily precipitation has the same monthly climatology and wet-day fractions with observations, but has its own wet-dry day sequence as produced by CMIP5 climate models. More details on the BCSD method can refer to Wood *et al*.^65^. In contrast to previous studies using GCM outputs directly^66^, runoff and soil moisture conditions are simulated by the Variable Infiltration Capacity (VIC) model^67, 68^ at the daily time step driven with the CMIP5 BCSD climate projections^69^ for 1950–2099 (hereafter referred to as CMIP5-VIC) and observed climate^39^ for 1950–2000 (hereafter referred to as OBS-VIC). The VIC model is a macroscale hydrologic model with representation of subgrid-scale variability^67, 68^ and has been widely used in investigating climate change impacts on droughts^26, 29, 30^. The VIC model settings including calibrated parameters and observed climate are based on Maurer *et al*.^39^. River routing is not considered in this study. Most of previous VIC applications used the first 1 year^70, 71^ or 3 years^72^ as model spinup period. Cosgrove *et al*.^73^ indicated that NLDAS models (including Mosaic, VIC, and Noah) can reach the equilibrium state within the first 1 to 2 years of simulations. Here, we conduct the analyses starting from 1971, discarding the first 21 years as the model spinup period.

### Definition of meteorological, agricultural, hydrological drought and the concurrence

In this study, the monthly meteorological, agricultural, and hydrological droughts are measured by the standardized precipitation index (SPI)^74, 75^, standardized soil moisture index (SSWI)^76^ and standardized runoff index (SRI)^31^, respectively. That is, meteorological drought is based on precipitation, while agricultural and hydrological drought is based on soil moisture and runoff, respectively. Here, the SPI is computed by fitting a gamma distribution to precipitation for specified months during the reference period 1971–2000. The gamma distribution is adopted since the distribution of monthly precipitation is typically similar to a gamma distribution. The SPI is designed with respect to normal conditions at a given site for a given period^74^. The calculation procedure for SRI and SSWI is similar to the SPI but by fitting a log probability density function to the runoff and soil moisture series^26^. To test the goodness-of-fit, the Kolmogorov-Smirnov (KS) nonparametric test is used (Supplementary Figure S4). A drought event is defined as the period of time when the value of drought index (i.e., SPI, SSWI and SRI) is below −1.0 corresponding to the probability of 6.7%, following the National Drought Mitigation Center (NDMC, http://drought.unl.edu). Concurrent drought is defined as the period when meteorological, agricultural and hydrological droughts are all experienced. In this study, we use the following three metrics for measuring droughts: (1) frequency: the number of drought events in a specific period; (2) duration: the length of a drought event; and (3) intensity: the average of index value lower than the drought threshold.

### Analysis

Daily precipitation, soil moisture and runoff are first temporally aggregated into monthly scale, and then spatially aggregated to the regional scale. Instead of defining regions with similar features^77^, we conduct our analysis for the HUC4 (i.e., 4-digit Hydrologic Unit Code) sub-basins (http://water.usgs.gov/GIS/huc.html), which is a simple average over its 1/8 degree grids. Here, the aggregated soil moisture for the three soil layers as represented in the VIC model are used. Meteorological, agricultural and hydrological droughts are derived based on the monthly time series of HUC4 sub-basin mean precipitation, soil moisture and runoff, respectively. Historical droughts based on CMIP5-VIC are validated against those by observation based OBS-VIC for the reference period 1971–2000. Future changes are calculated in a 30-year time window along 1971–2099 relative to the reference period 1971–2000 for each CMIP5-VIC combination under each RCP scenario. We acknowledge that the magnitude of changes may differ if a different present-day period is selected for analysis. The multi-model ensemble mean is used for illustration and the standard deviation among simulations is used to denote the uncertainty range from climate models^78^. Recently, the Paris agreement explicitly asks for assessments of the impacts of 1.5 °C global warming above preindustrial level^79^. Here, we conduct such assessments of 1.5 °C global warming on US droughts to provide the scientific basis for political discussions about mitigation and adaptation strategies. Specifically, global mean temperature is first calculated for each 30-year time window. We select the first 30-year period when the +1.5C global temperature is crossed for each climate model under each RCP. We then calculate the drought changes in the selected period corresponding to a 1.5C warming world for each model under each RCP. Investigation of drought changes with global warming can better inform policy-makers on regional mitigation and adaptation strategies, as governments and organizations are more concerned on the impacts associated with the target value of global mean temperature rise^79, 80^. In addition to climatological mean of droughts, we examine the changes in extreme droughts in terms of longest duration and largest intensity. What’s more, changes in droughts with four duration categories are calculated, i.e., D1, D2, D3 and D4 with duration of 1~3, 4~6, 7~12, >12 months, respectively. Specifically, we first calculate the changes in D1, D2, D3 and D4 droughts for each region. We then compare the changes among the four drought categories and select the one which shows the largest change than other three categories as the dominant one. The fraction of lands dominated by each drought category is then counted for each period to show the temporal evolutions.

## Electronic supplementary material

Supplementary Materials
