# Supplementary material for: The asymmetric impact of global warming on US drought types and distributions in a large ensemble of 97 hydro-climatic simulations
Source: Sci Rep. 2017 Jul 19;7:5891. doi: 10.1038/s41598-017-06302-z (PMC5517487; doi:10.1038/s41598-017-06302-z)
Supplement: Supplementary file 1 — Supplementary Materials [file 41598_2017_6302_MOESM1_ESM.pdf]

The asymmetric impact of global warming on drought types and distributions in the Conterminous United States in a large ensemble of 97 hydro-climatic simulations

Shengzhi Huang[1], Guoyong Leng[2,*],

Qiang Huang[1], Yangyang Xie[1], Saiyan Liu[1], Erhao Meng[1] and Pei Li[1]

[1]State Key Laboratory Base of Eco-Hydraulic Engineering in Arid Area, Xi'an University of Technology, Xi'an 710048, China

[2]Joint Global Change Research Institute, Pacific Northwest National Laboratory, College Park MD, USA

*Corresponding author address: Guoyong Leng, Joint Global Change Research Institute, Pacific Northwest National Laboratory, College Park MD, 20740.
E-mail: Guoyong.Leng@pnnl.gov

Supplementary Materials

Figure S1 Time evolution of US mean frequency, duration and intensity of meteorological, agricultural and hydrological droughts and the concurrent droughts under RCP2.6, RCP4.5, RCP6.0 and RCP8.5 scenarios. Solid lines are for multi-model ensemble mean while shaded areas are for one standard deviation denoting the model ranges. The year axis represents the 30-year period centered at that year. For example, the year 1985 indicate the period 1971-2000. Figure was created using software MATLAB 2015a (http://www.mathworks.com/).

Figure S2 Time evolution of the mean duration (blue) and longest duration (red) of meteorological, agricultural, hydrological and concurrent droughts under RCP8.5 scenario. Solid lines are for multi-model ensemble mean while shaded areas are for one standard deviation denoting the model ranges. The year axis represents the 30-year period centered at that year. For example, the year 1985 indicate the period 1971-2000. Figure was created using software MATLAB 2015a (http://www.mathworks.com/).

Figure S3 Same as Figure S2 but for drought intensity (unitless). Figure was created using software MATLAB 2015a (http://www.mathworks.com/).

Figure S4 Kolmogorov–Smirnov test results on the performance of log probability density function in deriving the SSI and SRI drought index. Blue color indicates the region where the log probability density function can’t fit the drought index at the 95% confidence level. Figure was created by NCAR Command Language[1]

Table S1 The ensemble of hydro-climate model projections used in this study

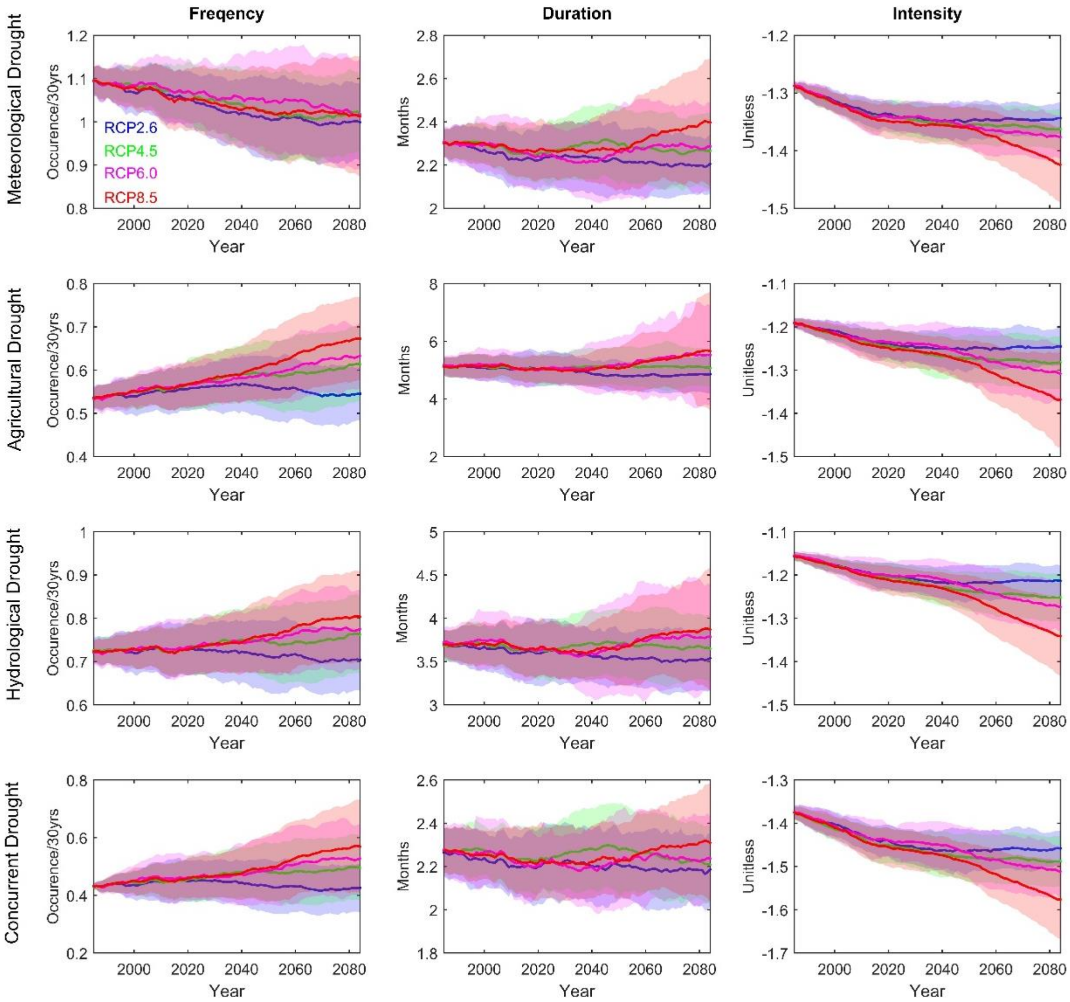

Figure S1 Time evolution of US mean frequency, duration and intensity of meteorological, agricultural and hydrological droughts and the concurrent droughts under RCP2.6, RCP4.5, RCP6.0 and RCP8.5 scenarios. Solid lines are for multi-model ensemble mean while shaded areas are for one standard deviation denoting the model ranges. The year axis represents the 30-year period centered at that year. For example, the year 1985 indicate the period 1971-2000. Figure was created using software MATLAB 2015a (http://www.mathworks.com/).

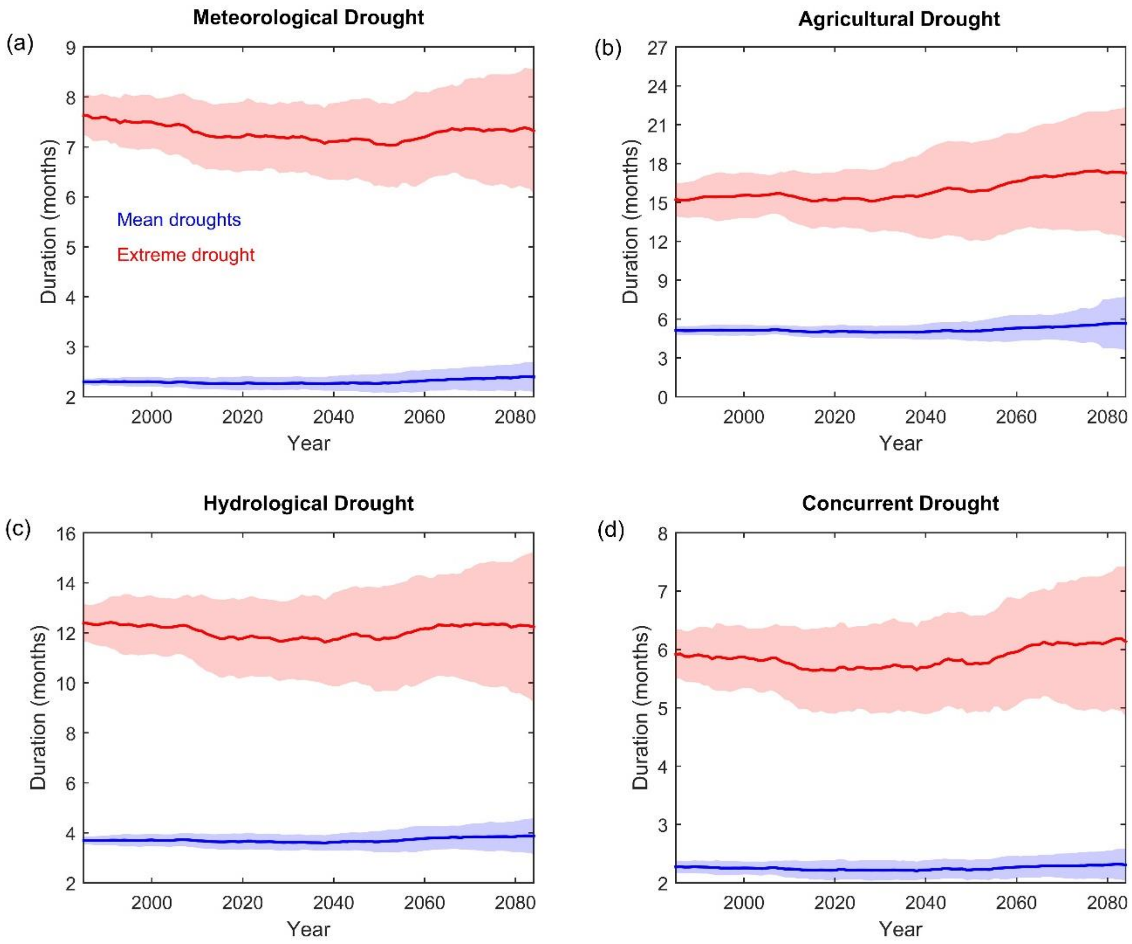

Figure S2 Time evolution of the mean duration (blue) and longest duration (red) of meteorological, agricultural, hydrological and concurrent droughts for 1971-2099 under RCP8.5 scenario. Solid lines are for multi-model ensemble mean while shaded areas are for one standard deviation denoting the model ranges. The year axis represents the 30-year period centered at that year. For example, the year 1985 indicate the period 1971-2000. Figure was created using software MATLAB 2015a (http://www.mathworks.com/).

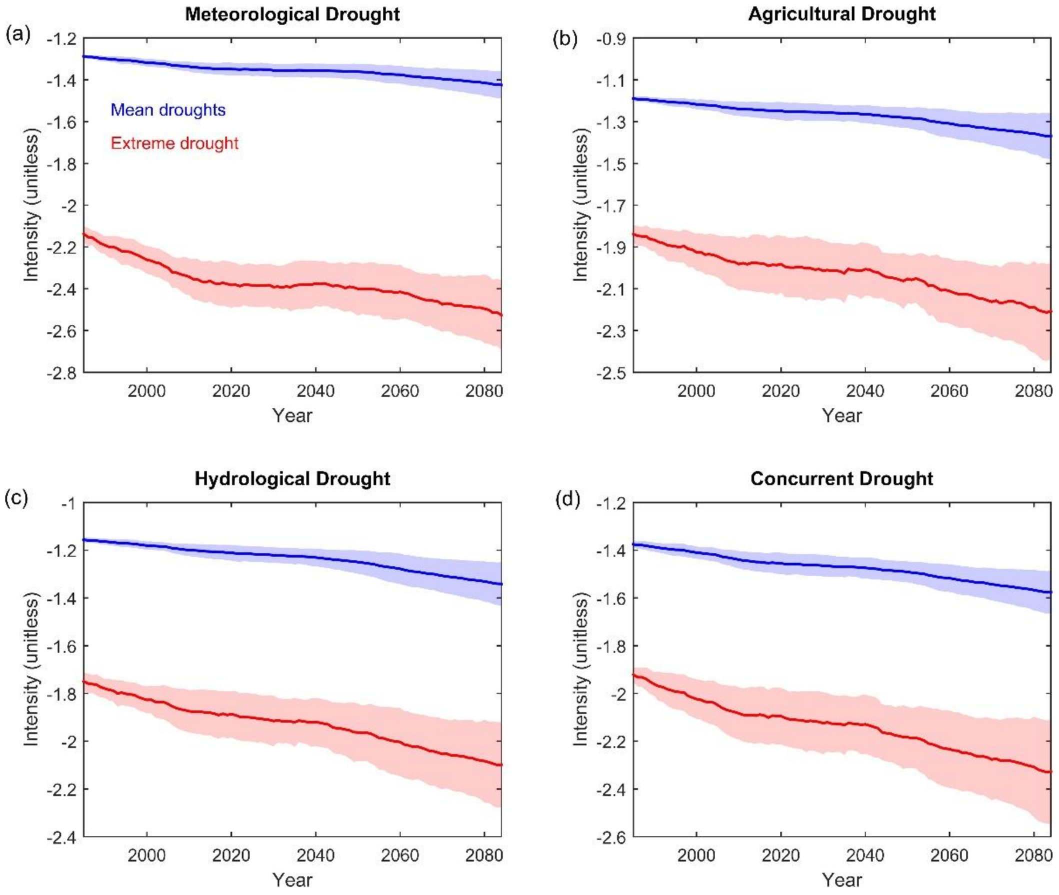

Figure S3 Same as Figure S2 but for drought intensity (unitless). Figure was created using software MATLAB 2015a (http://www.mathworks.com/).

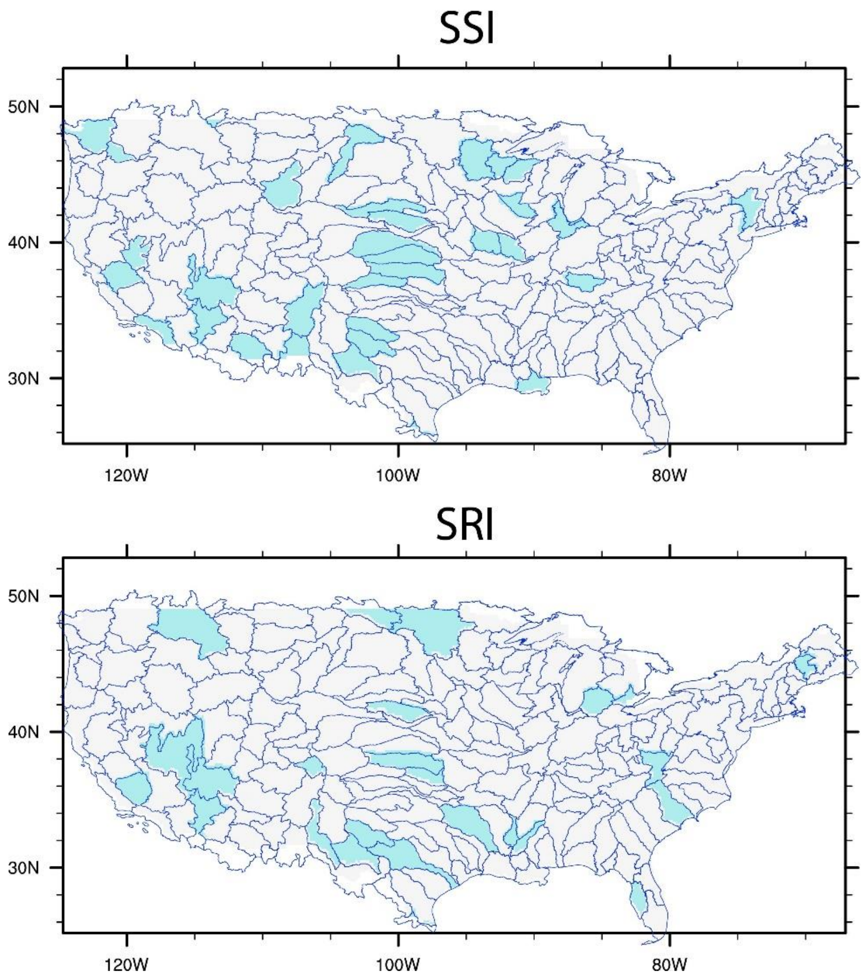

Figure S4 Kolmogorov–Smirnov test results on the performance of log probability density function in deriving the SSI and SRI drought index. Blue color indicates the region where the log probability density function can’t fit the drought index at the 95% confidence level. Figure was created by NCAR Command Language[1]

Table S1 The ensemble of hydro-climate model projections used in this study

ID	Climate Model	Emission Scenarios			
1	access1-0		rcp45		rcp85
2	bcc-csm1-1	rcp26	rcp45	rcp60	rcp85
3	bcc-csm1-1-m		rcp45		rcp85
4	canesm2	rcp26	rcp45		rcp85
5	ccsm4	rcp26	rcp45	rcp60	rcp85
6	cesm1-bgc		rcp45		rcp85
7	cesm1-cam5	rcp26	rcp45	rcp60	rcp85
8	cmcc-cm		rcp45		rcp85
9	cnrm-cm5		rcp45		rcp85
10	csiro-mk3-6-0	rcp26	rcp45	rcp60	rcp85
11	fgoals-g2	rcp26	rcp45		rcp85
12	fio-esm	rcp26	rcp45	rcp60	rcp85
13	gfdl-cm3	rcp26	rcp45	rcp60	rcp85
14	gfdl-esm2g	rcp26	rcp45	rcp60	rcp85
15	gfdl-esm2m	rcp26	rcp45	rcp60	rcp85
16	giss-e2-h-cc		rcp45		
17	giss-e2-r	rcp26	rcp45	rcp60	rcp85
18	giss-e2-r-cc		rcp45		
19	hadgem2-ao	rcp26	rcp45	rcp60	rcp85
20	hadgem2-cc		rcp45		rcp85
21	hadgem2-es	rcp26	rcp45	rcp60	rcp85
22	inmcm4		rcp45		rcp85
23	ipsl-cm5a-mr	rcp26	rcp45	rcp60	rcp85

24	ipsl-cm5b-lr		rcp45		rcp85
25	miroc-esm	rcp26	rcp45	rcp60	rcp85
26	miroc-esm-chem	rcp26	rcp45	rcp60	rcp85
27	miroc5	rcp26	rcp45	rcp60	rcp85
28	mpi-esm-lr	rcp26	rcp45		rcp85
29	mpi-esm-mr	rcp26	rcp45		rcp85
30	mri-cgcm3	rcp26	rcp45		rcp85
31	noresm1-m	rcp26	rcp45	rcp60	rcp85
Number of Hydrologic Projections	97	21	31	16	29

References

1. The NCAR Command Language (Version 6.1.2) [Software]. (2013). Boulder, Colorado: UCAR/NCAR/CISL/VETS. http://dx.doi.org/10.5065/D6WD3XH5.
